# Cardiovascular Effects of Dietary Salt Intake in Aged Healthy Cats: A 2-Year Prospective Randomized, Blinded, and Controlled Study

**DOI:** 10.1371/journal.pone.0097862

**Published:** 2014-06-18

**Authors:** Valérie Chetboul, Brice Stéphane Reynolds, Emilie Trehiou-Sechi, Patrick Nguyen, Didier Concordet, Carolina Carlos Sampedrano, Isabelle Testault, Jonathan Elliott, Jérôme Abadie, Vincent Biourge, Hervé Pierre Lefebvre

**Affiliations:** 1 Unité de Cardiologie d'Alfort, Centre Hospitalier Universitaire Vétérinaire d'Alfort, Université Paris-Est, Ecole Nationale Vétérinaire d'Alfort, Maisons-Alfort, France; 2 INSERM, U955, Equipe 03, Créteil, France; 3 Unité de Recherche Clinique, Université de Toulouse, INP, Ecole Nationale Vétérinaire de Toulouse, Toulouse, France; 4 Nutrition and Endocrinology Unit, LUNAM Université, Oniris, National College of Veterinary Medicine, Food Science and Engineering, Nantes, France; 5 UMR 1331 Toxalim, INRA, Université de Toulouse, INP, Ecole Nationale Vétérinaire de Toulouse, Toulouse, France; 6 Atlantia Veterinary Hospital, Nantes, France; 7 Department of Comparative Biomedical Sciences, Royal Veterinary College, Royal College Street, Camden, London, United Kingdom; 8 Department of Pathology LUNAM Université, Oniris, National College of Veterinary Medicine, Food Science and Engineering, Nantes, France; 9 Centre de Recherches, Royal Canin SAS, Aimargues, France; Medical University Innsbruck, Austria

## Abstract

High salt dry expanded diets are commercially available for cats to increase water intake and urine volume, as part of the prevention or treatment of naturally occurring urinary stone formation (calcium oxalates and struvites). However, chronic high salt intake may have potential cardiovascular adverse effects in both humans, especially in aging individuals, and several animal models. The objective of this prospective, randomized, blinded, and controlled study was to assess the long-term cardiovascular effects of high salt intake in healthy aged cats. Twenty healthy neutered cats (10.1±2.4 years) were randomly allocated into 2 matched groups. One group was fed a high salt diet (3.1 g/Mcal sodium, 5.5 g/Mcal chloride) and the other group a control diet of same composition except for salt content (1.0 g/Mcal sodium, 2.2 g/Mcal chloride). Clinical examination, systolic and diastolic arterial blood pressure measurements, standard transthoracic echocardiography and conventional Doppler examinations were repeatedly performed on non-sedated cats by trained observers before and over 24 months after diet implementation. Radial and longitudinal velocities of the left ventricular free wall and the interventricular septum were also assessed in systole and diastole using 2-dimensional color tissue Doppler imaging. Statistics were performed using a general linear model. No significant effect of dietary salt intake was observed on systolic and diastolic arterial blood pressure values. Out of the 33 tested imaging variables, the only one affected by dietary salt intake was the radial early on late diastolic velocity ratio assessed in the endocardium of the left ventricular free wall, statistically lower in the high salt diet group at 12 months only (P = 0.044). In conclusion, in this study involving healthy aged cats, chronic high dietary salt intake was not associated with an increased risk of systemic arterial hypertension and myocardial dysfunction, as observed in some elderly people, salt-sensitive patients and animal models.

## Introduction

On one hand, salt is a vital element for physiologic functions, including extra cellular fluid volume and blood pressure (BP) homeostasis, but on the other hand salt in excess may have potential deleterious cardiovascular effects [1]. Various experimental animal models, as well as human clinical trials and epidemiological studies, including the standardized worldwide INTERSALT Study, have provided evidence for a causal association between salt consumption and increase in BP values. However the results of these studies have been inconsistent and a marked variable individual salt sensitivity is evident, related in part to a genetic basis [2]–[5]. A high salt intake has also been demonstrated to be associated with myocardial function changes [6], [7] as well as increased left ventricular (LV) mass in both animal models [8], [9] and humans [10]–[12], independent of effects on BP.

Lower urinary tract diseases, such as urolithiasis and idiopathic cystitis, are common in the feline species [13]. One aspect of their long-term management is to increase water intake in order to subsequently increase urine volume and reduce urine solute concentration, which can be achieved by increasing dietary sodium [14]–[16]. Previous studies have shown the efficacy of appropriately designed high-salt dry diets to reduce struvite and calcium oxalate supersaturation (the most common minerals found in feline uroliths) and to dissolve naturally occurring feline struvite urinary stones [17], [18]. Therapeutic diets for cats with lower urinary tract diseases, characterized by a high salt content, are hence currently commercially available in order to enhance water intake and urine output. Several studies have already focused on the renal and cardiovascular safety of these high salt diets, and all reported the absence of significant adverse effect on systemic arterial BP, while significantly increasing water intake and decreasing urine specific gravity in comparison with cats fed a control diet [15], [16], [19], [20]. Nevertheless, none of the latter studies specifically focused on the potential deleterious effects of high-salt diets on global and regional myocardial function using sensitive imaging techniques such as tissue Doppler imaging (TDI). Additionally, these studies were all short- or medium-term feeding trials of 1-week to 6-month duration, performed on young (mean age 1 to 2.5 years old) to middle-aged adult cats (mean age of 7 years) only [15], [19], [20]. However aged cats are known to be at risk for both systemic arterial hypertension [21], [22] and chronic kidney diseases [23], two conditions that can be worsened by high-sodium diets in salt-sensitive humans and laboratory animals [24]–[27].

The objective of the present prospective, randomized, blinded, and controlled study was therefore to assess the long-term cardiovascular effects of dietary salt intake in healthy aged cats, using systemic arterial BP measurement, standard 2-dimensional (2D) and M-mode transthoracic echocardiography, conventional Doppler examination, and also 2D color TDI.

## Materials and Methods

### Animals

The present cardiovascular prospective study was performed concomitantly to another protocol, whose aim was to assess the effect of dietary salt intake on renal function using glomerular filtration rate (GFR) measurement, kidney ultrasonography, including renal resistive index assessment, and urinalysis [28]. Both inclusion and exclusion criteria used in the present study were identical to those of the latter protocol [28]. Briefly, 26 Domestic Shorthair neutered aged cats (10.1±2.4 years [5.3–14.5], 4.8±0.7 kg [3.6–6.5]) from a research colony housed in an indoor research facility with a 12 h light/dark cycle, controlled temperature (18–21°C) and ventilation (250 m^3^/h, 12 h/day) were screened for suitability for entry to the study. After baseline evaluations, cats were included in the study only if they were compliant for all scheduled procedures and if they were healthy on the basis of physical examination, BP measurement, routine urine and blood analyses, kidney ultrasonography, standard echocardiography, and conventional Doppler examination. According to the above-mentioned inclusion criteria, 20/26 healthy cats (10 males and 10 females; 10.1±2.4 years [5.5–11.7]; 4.8±0.7 kg [3.6–6.5]) were included in the study. Five out of the 26 cats from the research colony could not be included because of hyperthyroidism and chronic kidney disease (n = 1), chronic kidney disease (n = 1), hypertrophic cardiomyopathy (n = 1), chronic kidney disease and hypertrophic cardiomyopathy (n = 1), chronic liver disease (n = 1), and marked uncooperative behavior (n = 1). After group allocation, the 20 recruited healthy cats were allowed to acclimate with the other cats of their group for a 2 week-period, and were then regularly monitored over 2 years.

### Randomization procedure

To control the potential confounding effect of renal dysfunction on cardiac function and *vice versa*, a stratified randomization was performed. The 20 recruited healthy cats were first stratified in 3 subsets according to 2D color TDI results at baseline: normal (n = 8/20), subnormal (i.e., presence of regional post-systolic contraction waves for the left ventricular free wall (LVFW) and/or the interventricular septum (IVS) without any other alteration; n = 6/20), and abnormal (i.e., mild to moderate regional diastolic alterations characterized by an early on late diastolic velocity ratio (E/A ratio) <1; n = 6/20) [29]. The following randomization procedure was then performed separately within each subset (Table 1): cats were ranked according to their GFR and paired. In each pair of cats, the first was randomly assigned to one diet group and the second was assigned to the other diet group. This ensured that the cats in each diet group were well matched with regard to both renal and cardiac function. In addition, comparability of the 2 groups for all the variables of interest at baseline was assessed by use of Student's t-test. Any condition that could interfere with the study objective (occurrence of disease, need for treatment) or for which continuation of the study raised ethical concerns led to exclusion of affected cats.

**Table 1:** Final Group allocation for the 20 healthy cats included in the study.

Cat ID Code	TDI results	Gender	GFR (mL/min/kg)	Group
16	Normal	M	1.2	C
18	Normal	M	1.8	HS
7	Normal	M	1.8	C
13	Normal	F	1.8	HS
25	Normal	M	2.1	HS
23	Normal	F	2.1	C
14	Normal	F	2.5	HS
5	Normal	F	2.5	C
9	Subnormal	F	1.7	HS
21	Subnormal	F	1.9	C
4	Subnormal	M	1.9	C
6	Subnormal	F	2.0	HS
19	Subnormal	M	2.2	C
10	Subnormal	F	2.3	HS
8	Abnormal	M	1.6	C
17	Abnormal	M	1.6	HS
12	Abnormal	M	1.7	HS
24	Abnormal	F	1.8	C
11	Abnormal	M	1.9	HS
22	Abnormal	F	2.1	C

GFR, glomerular filtration rate; M, male; F, female; C, control group; HS, high salt group; TDI, tissue Doppler imaging.

Normal: normal systolic and diastolic radial and longitudinal myocardial function assessed by TDI.

Subnormal: normal systolic and diastolic radial and longitudinal myocardial function, except for the presence of regional post-systolic contraction waves for the longitudinal motion of the left ventricular free wall and/or the interventricular septum.

Abnormal: regional diastolic dysfunction (as assessed by an E/A ratio <1; E and A: early and late diastolic TDI waves).

### Diets

During screening, inclusion, group allocation and acclimation, cats were fed a maintenance dry expanded diet (*Veterinary Diet, Neutered Cats, Young Male, Royal Canin S.A.S., Aimargues, France*) with a sodium content of 0.7% as fed basis. After the acclimation period, cats were then monitored over 2 years while fed 70 g/day of either the high-salt diet (HSD, *Veterinary Diet, Feline Urinary High Dilution, Royal Canin S.A.S., Aimargues, France*, 1.3% sodium content and 2.27% chloride as fed basis) or the control diet (CD) of the same composition except for the level of sodium and chloride (0.35% sodium, 0.70% chloride) that was replaced with corn flour (Table 2). The level of salt chosen for the control diet is that commonly found in commercial dry cat foods. The food amount (70 g/day) was arbitrarily selected to be greater than the usual consumption of these cats. Food leftovers were weighed and each cat's exact food intake recorded daily. Cats had also free access to water.

**Table 2:** Nutrient composition of the diets used in the study.

Nutrient (g/Mcal ME)	HSD	CD
Moisture	13.6±0.8	16.0±1.5
Proteins	87.0±3.8	84.0±2.8
Fat	39.2±1.8	39.5±1.5
Minerals	21.1±1.3	15.3±0.3
Total Dietary Fiber	16.1±2.0	18.0±2.3
Sodium	3.1±0.1	1.0±0.1
Chloride	5.5±0.3	2.2±0.3
ME (kcal/kg, NRC 2006)	3976±55	4000±32

CD: control diet; HSD: High salt diet (Veterinary Diet Urinary High Dilution, Royal Canin, Aimargues, France); ME: Metabolizable energy; NRC: National Research Council.

### Experimental design

The protocol was reviewed and approved by the animal care and use committee of “Pays-de-la-Loire” (file n°CEEA.2012.152) local as well as the Royal Canin ethic committee. This study was conducted according to conditions approved by the French Ministry of Agriculture and to the guidelines of the Guide for Care and Use of Laboratory Animals [30]. Physical examination, systolic and diastolic arterial BP, standard echocardiography, and conventional Doppler examination as well as 2D color TDI were performed at baseline and at 6, 12, and 24 months. Physical examination and BP measurements were also performed at 3 months, and body weight was assessed weekly throughout the study. Investigators were blinded to the treatment groups and cats were randomly ranked for iterative measurements (i.e., physical examination, BP measurements, conventional ultrasound examinations, and TDI) at each follow-up point.

### Blood pressure measurement

Systolic and diastolic systemic arterial BP was measured indirectly in awake cats by the same trained observers (CCS, ET) by use of a Doppler system (*811-BL Parks Medical Electronics Inc, ALOHA, OR*), as previously described [31] and according to the guidelines currently recommended for the feline species [22]. Systemic arterial hypertension was defined as BP >160 (systolic) and/or >100 (diastolic) mmHg [22].

### Standard echocardiography and conventional Doppler examination

Standard transthoracic echocardiography and conventional Doppler examination were performed by a single trained observer (VC) using an ultrasound unit (*Vivid 7 dimension, General Electric medical system, Waukesha, Wis, USA*) equipped with 2 phased-array transducers 7S (3.5–8 MHz) and 10S (4.5–11.5 MHz). All ultrasound examinations were carried out with continuous electrocardiogram (ECG) monitoring in awake cats, gently restrained in the standing position as already described and validated by our group [32]. A mean of 3 measurements was obtained for each M-mode parameter on 3 consecutive cardiac cycles on the same frame. Left ventricular (LV) diameters, LVFW and IVS thicknesses were measured at end-diastole and end-systole from the right parasternal short-axis view [33] by use of the 2D-guided M-mode according to the recommendations of the American Society of Echocardiography [34], and the LV shortening fraction was then calculated. Measurements of the aorta (Ao) and the left atrial (LA) diameter were obtained by a 2D method at end-diastole, and the LA/Ao ratio was then calculated [32]. The end-diastolic subaortic IVS thickness was also measured using a 2D method from the right parasternal 5-chamber view at the level of the attachments of the left *chordae tendineae* to the mitral valve leaflets, as previously described [31]. The presence of a systolic anterior motion of the mitral valve, defined as a motion of the anterior mitral valve leaflet towards the LV outflow tract, was also assessed using both 2D and M-modes. Lastly, maximal systolic aortic velocity and maximal early and late diastolic mitral flow velocities (mitral E and A waves, respectively) were determined using the pulsed-wave Doppler mode from the left apical 5- and 4-chamber views, respectively, and the mitral E/A ratio was then calculated. The isovolumic relaxation time (IVRT, time interval between end of aortic flow velocity and onset of transmitral flow) was also calculated from the left apical 5-chamber view using the pulsed-wave Doppler mode. Echocardiographic and Doppler examinations were considered as normal if the latter 2D, M-mode, and Doppler variables were within the reference ranges published by our group [29].

### Tissue Doppler imaging examination

**Figure 1:**
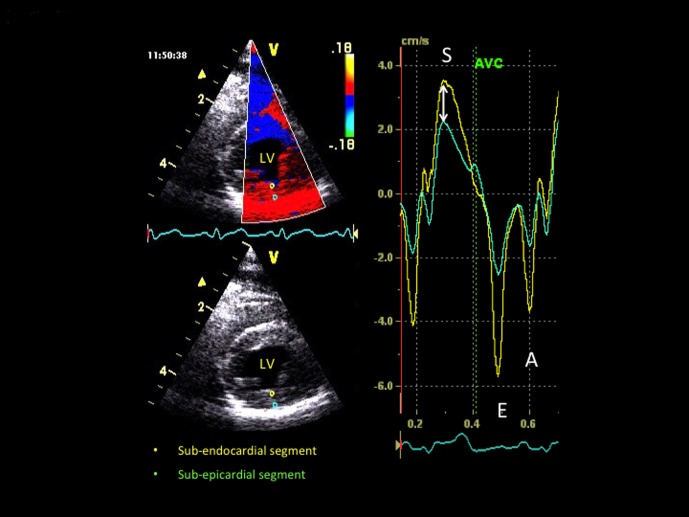
Radial velocity profiles obtained in a healthy recruited cat by two-dimensional color tissue Doppler imaging from the right parasternal transventricular short-axis view, simultaneously in a sub-endocardial (yellow) and a sub-epicardial (green) segment of the left ventricular free wall. *S, E and A: peak myocardial velocity during systole, early diastole and late diastole, respectively. AVC: aortic valve closure. Double arrow: systolic myocardial velocity gradient. LV: left ventricle.*

**Figure 2:**
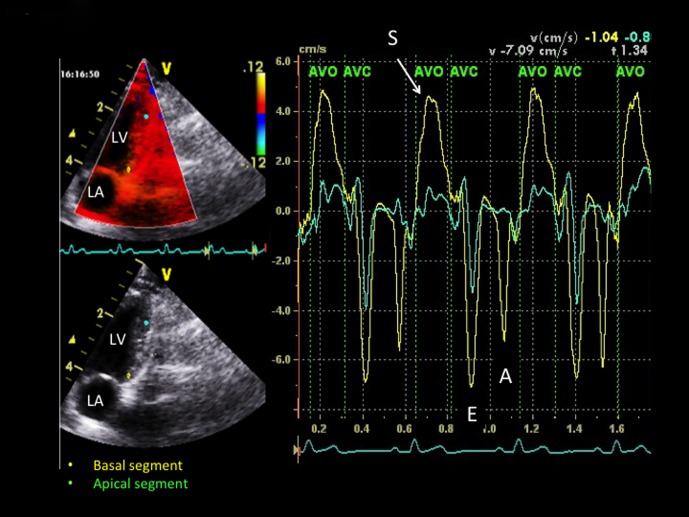
Longitudinal velocity profiles obtained in a healthy recruited cat by two-dimensional color tissue Doppler imaging from the left apical 4-chamber view, simultaneously in a basal (yellow) and apical (green) segment of the left ventricular free wall. *S, E and A: peak myocardial velocity during systole, early diastole and late diastole, respectively. AVO and AVC: aortic valve opening and aortic valve closure, respectively. LA: left atrium. LV: left ventricle.*

All 2D color TDI examinations were performed and interpreted in awake standing cats with continuous ECG monitoring by the same single observer (VC) and using the same ultrasound unit as for standard echocardiography, as previously described and validated [35]. Real-time color Doppler was superimposed on the gray scale with a high frame rate (between 180 and 280 frames/s). The Doppler receive gain was adjusted to maintain optimal coloring of the myocardium (i.e., without any black spots), and the Doppler velocity range was set as low as possible to avoid aliasing. All digital images were stored and analyzed using specific software (*Echopac Dimension, General Electric Medical System, Waukesha, Wisc, USA*). A 1×1 mm sample was used and a tissue velocity profile displayed in each sample location. Peak myocardial velocities resulting from radial LVFW motion were measured in systole, early and late diastole (S, E and A waves) using the right parasternal ventricular short-axis view, and measurements were made between the 2 papillary muscles in sub-endocardial and sub-epicardial segments of the LVFW (Figure 1). Peak systolic (S), early (E) and late (A) diastolic longitudinal velocities were also measured using the standard left apical 4-chamber view in 3 myocardial segments, i.e., 2 from the LVFW (at the base and the apex, Figure 2) and 1 from the IVS (at the base). The TDI diastolic E/A ratio was calculated for each of these 5 myocardial segments. Radial systolic myocardial velocity gradients (MVG, defined as the difference between sub-endocardial and sub-epicardial systolic velocities) and longitudinal systolic MVG (defined as the difference between basal and apical systolic LVFW velocities) were also calculated for each phase of the cardiac cycle. Lastly, mean heart rate was calculated by ECG monitoring during each radial and longitudinal TDI examination from the same 3 cardiac cycles used for velocity measurements.

### Statistical analysis

Data are expressed as mean ± standard deviation. Time course of body weight was analyzed by a linear mixed effects model incorporating diet, time and diet by time interaction as fixed effects, cats within diet and cats by time interaction as random effects with a software (*R Development Core Team (2009). R: A language and environment for statistical computing. R Foundation for Statistical Computing, Vienna, Austria. ISBN 3-900051-07-0, URL* http://www.R-project.org). For other variables, standard repeated measures analyses were performed with another software package (*Systat version 8.0, SPSS Inc, Chicago, IL, USA*) by use of the following generalized linear model:
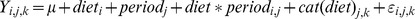



With:

Y_i,j,k_ being the value of variable Y for Cat k with diet i in Period j 

µ being the general mean effect 

diet_i_ being the effect of diet (i  =  HSD or CD) 

period_j_ being the effect of period (j = 0, 3, 6, 12 or 24 months) 

diet*period_i, j_ being the diet by period interaction term of the model 

cat(diet) _j, k_ being the effect of cat nested in its diet group 

ε_i, j_, k being the error of the model.

Effect of dietary salt intake on tested variables was primarily assessed through the diet by period interaction term of the model. Whenever a significant diet by period interaction was detected, results of cats from the HSD group were compared to those from the CD group at each period by use of a Student's t-test. A value of P<0.05 was considered significant.

## Results

### Study feline population and follow-up

All recruited cats (n = 20) completed the first 12-month period and 16/20 the 24-month follow-up, as 4 cats were removed from the study between 12 and 24 months (2 from each diet group, i.e., cats #11, #16, #18 and #24, see Table 1). Cats #11 and #18 from the HSD group died suddenly at 13 and 21 months from no obvious cause and intracranial meningioma at full necropsy, respectively. Cat #24 from the CD group was euthanized at 13 months because of cancer (fibrosarcoma) and cat #16 from the same group was removed from the study at 17 months because of occurrence of diabetes mellitus. Analysis of the diets confirmed that, apart from salt content, differences between the 2 diets were negligible and could not interfere with the study objective. Mean caloric intake over the study period was 46±11 kcal ME/kg/day for the HSD group and 48±6 kcal ME/kg/day for the CD group, representing a dietary sodium intake of 144±36 and 45±5 mg/kg/day, respectively.

### Diet effects on physical examination

All cats that completed the study remained healthy throughout the first 12-month period (n = 20) and between 12 and 24 months (n = 16), without any sign of congestive heart failure. Cardiac auscultation did not reveal any arrhythmia. Heart rate was comparable at baseline between the 2 groups (167±18 bpm and 186±25 bpm for the CD and HSD groups, respectively), and remained stable over the 24-month period. A mild but significant (P = 0.043) decrease in body weight of approximately 120 g/year was similarly observed in both diet groups during the study period (from 4.8±0.7 kg on the first day of the diet test period to 4.5±0.8 kg after 2 years in the whole study population).

### Diet effects on systolic and diastolic BP

Systolic as well as diastolic BP values were comparable at baseline between the CD and HSD groups (i.e., 149±6 mmHg and 153±3 mmHg in systole, 78±8 mmHg and 78±11 mmHg in diastole, respectively). No systemic arterial hypertension was found in any cat throughout the 24-month study period, and no significant effect of the diet composition was found during the whole study period.

### Diet effects on 2D and M-mode echocardiographic variables

All 2D and M-mode echocardiographic variables (n = 9) assessed at baseline were similar between the two groups (Table 3) and remained within reference intervals throughout the study for all cats [29]. No systolic anterior motion of the mitral valve, leading to LV outflow tract obstruction, was detected in any cat using both 2D and M-modes. No significant statistical effect of diet composition was found on any of the tested echocardiographic variables.

**Table 3:** Effects of dietary salt content on conventional echocardiographic and standard Doppler variables (means ± SD) assessed in healthy aged cats fed a high salt diet (HSD, n = 10) or a control diet (CD, n = 10) over 24 months.

Imaging variables	0		6 months		12 months		24 months	
	CD	HSD	CD	HSD	CD	HSD	CD	HSD
***M-mode variables***								
LVDd (mm)	14.5±1.3	13.8±1.2	14.4±2.1	13.9±0.7	15.0±1.1	14.2±1.4	13.9±1.4	13.7±1.0
LVDs (mm)	6.7±0.9	6.7±1.3	6.7±1.3	6.6±1.1	6.9±1.4	6.1±1.0	5.9±1.3	6.0±1.0
LVFWd (mm)	4.6±0.3	4.3±0.4	4.5±0.3	4.3±0.4	4.5±0.3	4.5±0.4	4.5±0.5	4.2±0.4
LVFWs (mm)	7.9±0.7	7.5±0.8	7.9±0.8	7.7±0.9	8.2±0.6	8.0±0.9	8.6±1.0	7.9±1.0
IVSd (mm)	4.8±0.4	4.8±0.5	4.8±0.4	4.7±0.4	4.8±0.4	4.8±0.5	4.6±0.4	4.3±0.4
IVSs (mm)	7.8±0.9	7.7±0.7	8.1±1.1	7.8±0.7	8.1±0.7	8.0±0.7	8.3±0.8	7.7±0.7
Fractional shortening (%)	53.9±6.2	51.5±7.3	53.6±6.0	52.7±7.3	54.6±7.3	57.3±5.1	57.9±7.0	56.3±7.7
***Two-dimensional variables***								
Left atrium/aorta	0.84±0.08	0.83±0.07	0.81±0.09	0.75±0.12	0.87±0.14	0.82±0.14	0.78±0.09	0.75±0.12
Subaortic IVSd (mm)	4.4±0.6	4.5±0.5	4.5±0.6	4.5±0.5	4.6±0.5	4.5±0.5	4.6±0.5	4.4±0.3
***Doppler variables***								
Peak aortic flow velocity (m/s)	1.2±0.3	1.2±0.2	1.1±0.2	1.1±0.1	1.2±0.2	1.2±0.2	1.0±0.2	1.1±0.2
Mitral E wave/A wave ratio	1.5±0.3	1.5±0.7	1.5±0.3	1.6±0.9	1.3±0.2	1.4±1.0	1.2±0.2	1.3±0.5
Isovolumic relaxation time (ms)	49±7	48±12	46±4	45±7	47±6	51±5	50±8	52±6

LVDd: Left ventricular end-diastolic diameter. LVDs: Left ventricular end-systolic diameter. LVFWd: left ventricular free wall at end-diastole. LVFWs: left ventricular free wall at end-systole. IVSd: interventricular septum at end-diastole. IVSs: interventricular septum at end-systole.

### Diet effects on conventional Doppler variables

The 3 tested conventional Doppler variables assessed at baseline were comparable between the CD and HSD groups (Table 3), and remained within reference intervals throughout the 24-month study period for all cats [29]. None of them was affected by the diet.

### Diet effects on radial and longitudinal systolic and diastolic 2D color TDI variables

Radial and longitudinal 2D color TDI variables (n = 11) were comparable at baseline between the CD and HSD groups (Table 4). A significant diet by period interaction over the study period was observed for the TDI E/A ratio measured in the sub-endocardial segment (P = 0.009). When compared at each period, this ratio was significantly different between groups at 12 months only (1.7±0.3 and 1.4±0.4 for the CD and HSD groups, respectively; P = 0.044). Other TDI variables were not affected by the diet.

**Table 4:** Effects of dietary salt content on radial and longitudinal tissue Doppler imaging (TDI) variables (means ± SD) assessed in healthy aged cats fed a high salt diet (HSD, n = 10) or a control diet (CD, n = 10) over 24 months.

TDI variables	0		6 months		12 months		24 months	
	CD	HSD	CD	HSD	CD	HSD	CD	HSD
***Radial motion of the left ventricular free wall***								
Heart rate (beats/min)	177±18	170±12	181±22	180±15	166±18	178±12	167±13	176±17
Systolic radial MVG (cm/s)	2.4±0.7	2.1±0.6	2.7±0.7	2.1±0.7	2.5±0.6	2.5±0.6	2.7±0.6	2.7±0.8
E/A ratio endocardium	1.4±0.4	1.7±0.5	1.8±0.4	2.0±0.6	**1.7±0.3**	**1.4±0.4** *	1.7±0.7	1.3±0.6
E/A ratio epicardium	1.6±0.7	2.0±1.4	2.5±1.3	2.4±1.2	2.4±1.2	1.7±0.6	2.9±2.3	1.7±1.2
***Longitudinal motion of the left ventricular free wall***								
Heart rate (beats/min)	178±19	165±23	183±23	187±20	170±16	180±16	174±31	177±17
Systolic MVG base-apex (cm/s)	2.2±0.9	2.5±1.3	2.4±0.7	2.3±1.2	2.3±0.7	2.5±1.1	2.4±0.6	2.6±0.8
E/A ratio at the base	1.8±1.5	1.5±0.5	1.4±0.5	1.7±0.9	1.5±0.7	1.6±0.8	1.2±0.5	1.2±0.5
E/A ratio at the apex	2.8±2.7	4.3±6.6	2.1±1.5	2.9±1.8	2.5±2.3	3.0±1.7	5.2±7.6	2.3±1.6
***Longitudinal motion of the interventricular septum***								
Heart rate (beats/min)	176±34	175±16	186±26	185±17	180±25	184±19	174±24	189±15
S wave at the base (cm/s)	5.5±2.0	5.8±1.6	5.7±1.8	6.0±1.5	6.0±1.8	6.4±1.4	6.1±1.7	6.5±1.1
E/A ratio at the base (cm/s)	1.0±0.5	1.2±0.6	1.2±0.5	1.3±0.7	1.1±0.5	1.1±0.4	1.1±0.5	0.9±0.5

S, E and A: peak myocardial velocity during systole, early diastole and late diastole, respectively. MVG: myocardial velocity gradient.

*P = 0.044 versus CD group.

## Discussion

In the present prospective study, BP, heart rate, cardiac morphology as well as myocardial function remained unaffected in healthy aged cats fed a HSD (1.3% sodium content and 2.27% chloride as fed) for 24 months, as compared with those fed a CD similar in all respects except for the salt content (0.35% sodium, 0.70% chloride).

The present protocol has several major key features: the study was prospective, controlled, blinded, randomized, and performed over a long-term period (24 months *versus* maximum 6 months in other feline studies on the topic [15], [16], [19], [20]). Moreover, for conventional echocardiography and TDI examinations, only one single trained experienced observer was involved, thus limiting the variability for the assessed imaging measurements to intra-operator variation [32], [35]. Additionally, cats in each diet group were deliberately matched with regard to both renal and cardiac function, as respectively assessed by GFR and TDI examination. This was of particular importance as, on the one hand, renal function can be altered in feline heart diseases [36] and, on the other hand, cats with chronic kidney diseases can undergo changes in cardiac morphology and function, partly due to systemic arterial hypertension that is commonly associated with chronic kidney disease in this species [22], [31]. Lastly, aged cats with a mean age of 10 years (only 1 cats/group were less than 7 *(i.e:5.3 yr)* years of age) were deliberately recruited, as old cats are likely to be at higher risk than younger cats for spontaneous systemic arterial hypertension and chronic kidney diseases [21]–[23], both of which are known to be worsened by high salt intake in human patients and laboratory animals [24]–[27]. Additionally, 1) BP has been shown in some studies to increase with age in the feline species [22], 2) a significant positive relationship between salt intake and the slope of the rise in BP with age has been reported in humans [27], and lastly, 3) age-related increase in salt sensitivity, although not demonstrated in the cat, is well recognized in humans, resulting at least in part, from the impairment of several mechanisms involved in sodium regulation, including a reduced ability to appropriately excrete a salt load owing to a decline in renal function and reduced generation of natriuretic substances, such as prostaglandin E2 and dopamine [27], .

Although the topic still remains debated and controversial in human medicine [38]–[41], there is substantial evidence supporting the deleterious effects of high consumption of salt on health, particularly regarding the cardiovascular system. For example, many studies showed a significant causal relationship between high salt intake and the development of systemic arterial hypertension in salt-sensitive patients and laboratory animals, and raised BP is known to be a major independent risk factor of cardiovascular diseases [1]–[3], [25]–[27], [37], [42]. Conversely, as recently shown by high quality evidence, a reduction in salt intake decreases BP in both hypertensive and normotensive individuals, and is associated with a reduced risk of stroke and fatal coronary heart disease [43]–[46]. Most international guidelines recommend therefore restricting salt intake in people [26], [27], [47], [48]. Several mechanisms by which high sodium intake diets can promote the development of hypertension have been reported, including changes in vascular reactivity, the renin-angiotensin-aldosterone system, and sympathetic reflexes [25], [49], [50], [51]. All these data led us to measure BP in all animals throughout the study period at Day 0 and then at 3, 6, 12, and 24 months in the present study, in order to assess the cardiovascular safety of one of the HS diets commercially available for cats with lower urinary tract diseases. No systemic arterial hypertension was found in any cat throughout the 24-month study period. No significant effect of the diet was detected either. The positive above-mentioned relationship between salt intake and the slope of the rise in BP with age reported in humans [27] was hence not found in these aged cats, which do not seem therefore to be sensitive to the deleterious vascular effects of excess dietary salt intake as observed in elderly people. These results are in accordance with those reported in this species in short- and medium-term feeding trials, showing that high salt feeding (2.9 to 3.2 g Na/Mcal) does not affect BP in healthy cats [15], [16], [19], [20].

In addition to its influence on BP, dietary sodium may exert several non-blood pressure-related effects, which result in direct target-organ damage, including myocardial hypertrophy and fibrosis as well as alteration of myocardial function, thus amplifying the effect of BP on the LV. These BP-independent cardiac adverse effects have been demonstrated in animal models [8], [9] and in both normotensive people and patients with essential hypertension [10]–[12], [24], [52], [53]. For example, in normal mice, chronic excess salt intake has been shown to induce a IVS-predominant LV hypertrophy, associated with an increase in collagen density, angiotensin converting enzyme activity, angiotensin II type 1 receptor density, and extracellular signal regulated kinase phosphorylation. All the latter data regarding the BP-independent cardiac adverse effects led us to perform a conventional echocardiographic examination on all animals throughout the present study (at Day 0 and then at 6, 12, and 24 months). Nevertheless, no significant statistical effect of diet composition was found on either 2D or M-mode echocardiographic variables, including myocardial wall thicknesses and LV diameters. Additionally, neither obstruction of the LV outflow tract (as confirmed by maximal systolic aortic velocity measurement) nor systolic anterior motion of the mitral valve (a common cause of dynamic obstruction of the LV outflow tract in cats with hypertrophic cardiomyopathy [54]) were detected, and the LA/Ao ratio remained unchanged over the whole study period. In other words, the 24-month diet was not associated with diffuse or localized myocardial hypertrophy, changes in LV diameters, and left atrial dilation.

As high salt diets have also been shown to modulate and affect myocardial function, particularly during the diastolic time [6], [7], [55], and as feline systemic arterial hypertension is associated with myocardial dysfunction occurring independently of the presence of myocardial hypertrophy [56], another aim of the present study was to assess the effect of high salt intake on myocardial function in the recruited aged cats. For this purpose, 2D color TDI, which has been shown by our group to be repeatable and reproducible in the awake cat [35], was chosen to complement the conventional echocardiographic and Doppler data and accurately analyze the effect of HSD on segmental myocardial function. We have previously demonstrated that 2D color TDI is more sensitive than conventional ultrasound techniques in detecting myocardial dysfunction in the feline species, even in the absence of overt myocardial changes [56]–[58]. As feline spontaneous hypertrophic cardiomyopathy and chronic systemic hypertension (SHT) can both lead to radial and longitudinal diastolic myocardial alteration and can both induce a longitudinal-predominant systolic LVFW dysfunction [56]–[58], both radial and longitudinal LV motions were assessed using 2D color TDI in the present study. Nevertheless, the 24-month HSD diet was not associated with overt systolic and diastolic dysfunction, as respectively the shortening fraction and both mitral E/A ratio and IVRT remained unchanged throughout the study. Similarly, regional radial and longitudinal systolic myocardial function, as assessed by TDI systolic velocity gradients (respectively, between sub-endocardial and sub-epicardial segments, and between basal and apical segments) remained unaltered throughout the 24-month study period. The 24-month HS diet had no effect either on the longitudinal diastolic function, as assessed by the longitudinal TDI E/A ratio in basal and apical segments of the LVFW as well as at the base of the IVS. Similarly, no difference according to dietary salt intake in the radial TDI E/A ratio repeatedly measured in the sub-endocardial and sub-epicardial segments of the LVFW over 24 months was found in the cats of the present study, except for the sub-endocardial TDI E/A ratio that was significantly lower in the HSD group (1.4±0.4) than in the CD group (1.7±0.3) at 12 months. Nevertheless, this 0.3 mean difference can be considered as biologically negligible [29], [35], and was detected only one time (at 12 months), and therefore cannot be interpreted as a long-term consistent adverse effect of HSD on sub-endocardial radial diastolic function. Additionally, given the number of TDI measurements that were compared during the whole study period, it is highly likely that this sole difference was detected just by chance.

In conclusion, in the present study, high dietary salt intake (3.1 g Na/Mcal) over 24 months had no adverse effects on BP, heart rate, cardiac morphology as well as global and regional myocardial function as assessed by 2D and M-mode echocardiography, conventional Doppler examination, and 2D color TDI in healthy aged cats. However, these results cannot be generalized to diseased cats suffering from spontaneous systemic arterial hypertension, cardiomyopathies, or chronic kidney disease. Additionally, further studies are needed to confirm this lack of salt-sensitivity in larger healthy populations of various feline breeds and mixed-breeds (Domestic short and long haired cats), and to understand the underlying mechanisms of this potential species specificity.
